# An analysis of weight loss articles and advertisements in mainstream women’s health and fitness magazines

**DOI:** 10.15171/hpp.2016.14

**Published:** 2016-06-11

**Authors:** Danna Ethan, Corey H. Basch, Grace Clarke Hillyer, Alyssa Berdnik, Mary Huynh

**Affiliations:** ^1^Department of Health Sciences, Lehman College, the City University of New York, New York, USA; ^2^Department of Public Health, William Paterson University, New Jersey, USA; ^3^Department of Epidemiology, Mailman School of Public Health, Columbia University, New York, USA

**Keywords:** Weight loss, Women's magazines, Advertisements, Articles

## Abstract

**Background:** Magazines are a commonly used source for health and fitness information. Little is known about the nature and extent of weight loss strategies and products presented in mainstream women’s health and fitness magazines.

**Methods:** This preliminary cross-sectional study evaluated the prevalence of articles and advertisements featuring weight loss content and products in mainstream US-based health and fitness magazines, as well as assessed weight loss themes presented. Thirty-one US health and fitness-focused magazine issues were coded. Prevalence of, product type, and themes related to weight loss in articles and advertisements were assessed.

**Results:** Among the 31 issues of the five US-based women’s magazines examined, we identified 39 articles (4.8% [95% CI = 3.3% to 5.5%] of 819 articles) related to weight loss with 14 identified weight loss topics. The most prevalent article topics covered were exercising/workouts (32.0% [95% CI = 28.8% to 33.6%]) followed by dieting (18.6% [95% CI = 15.9% to 19.9%]).The most common product advertised was weight loss pills (46.0% [95% CI = 42.6% to 47.7%]). Fat burners were also frequently advertised (14.9% [95% CI = 12.5% to 16.1%]) followed by hunger reduction strategies (10.3% [95% CI = 8.2% to 11.3%]) and fat blockers (6.9% [95% CI= 5.2% to 7.8%]).

**Conclusion:** Articles presented information about exercise and dieting whereas advertisements supported potentially harmful health beliefs and behaviors. As a well-utilized American media format, health and fitness-focused magazines have an opportunity to communicate frequent,accurate messaging about healthy weight reduction and limit advertisements that may include misleading claims.

## Introduction

Current statistics indicate that almost two-thirds of American women are overweight or obese.^1^ Consequently, it is estimated that the annual expenditure on weight loss products is 33 billion dollars in the United States.^2^ The growing weight loss industry is comprised of various services, venues and products including the diet soda market, health clubs, commercial weight loss chains, books and DVDs on exercise and various diets, medical weight loss programs, pills, foods and other products that claim to facilitate weight loss.^3,4^ Charged with protecting consumers from deceptive marketing practices, the Federal Trade Commission (FTC) has indicated there may be hundreds of weight loss products available and advertised on a wide variety of platforms such as magazines, television, and newspapers.^5^ In an analysis of these advertisements conducted by the FTC, 40% were found to include claims that were either false or misleading.^5^

Magazines, specifically, are a well-utilized source of health and nutrition information.^6-8^ In a literature review by Cutilli, findings indicate that health information-seeking behaviors often include referencing magazines.^6^ The study calls for nurses to be aware of these behaviors to improve the health of their patients.^6^ Maibach and colleagues concluded in their study that traditional media sources like magazines were a preferred medium for seeking health information.^7^ And, in a study using Health Styles data, Dutta-Bergman suggested that those who are considered more health-oriented are likely to access health information via active communication channels including print sources.^8^

A number of studies have examined the effects of magazine readership on outcomes such as body image and disordered eating habits and suggest that triggers in editorial content and advertisements can adversely affect behavior and health.^9-12^ For instance, body types utilized in editorial content and advertisements have been shown to influence adult and adolescent females’ body image and eating habits. One study found that women who were shown advertisements with thin models had more weight-related anxiety about their appearance and body dissatisfaction than those in the control group.^10^ Female participants in a separate study were assessed for their levels of exposure to magazines and television. While both media channels were associated with body dissatisfaction, only magazines were directly associated with the internalization of thin ideals.^11^ Turner and colleagues focused their study on determining whether fashion magazine exposure influenced body image. They found that those who viewed fashion magazines reported less satisfaction with their weight and their bodies than those who viewed the news articles.^12^

To date, the majority of print-based studies on this topic have focused on fashion magazines. Fewer studies have examined exclusively health and fitness-focused magazines, and while many have assessed the appearance of models and body satisfaction, little research has examined themes presented in editorial content and advertisements for weight loss strategies and products. Thomsen et al found that increased frequency of reading health and fitness magazines was associated with eating disordered cognition and behaviors in adolescent girls.^13^ In a qualitative analysis of young female athletes, the poses of models in health, fitness, and sports magazines that emphasized aesthetic beauty were associated with a negative self-evaluation of body image.^14^ Willis and Knobloch-Westerwick examined editorial content from popular women’s magazines and found that more than one-fifth of the articles addressed body shaping or weight loss.^15^ Although the majority of the editorial content promoted healthy behaviors such as exercising to lose weight and reducing caloric intake, the motivations utilized to encourage these behaviors were appearance-based. Women who are motivated by appearance are more likely to engage in unhealthy weight loss behaviors.^16^

There is also a dearth of literature that has examined themes found in weight loss product advertisements and their influence on health beliefs and behaviors. A 2003 study by Geier and colleagues found that advertisements including “before” and “after” pictures were associated with anti-fat bias and beliefs that weight is easily controllable.^17^ Purchasing weight loss products can be driven by health-oriented motivators (e.g. health benefits associated with weight loss); however, some consumers may be motivated by the desire for a ‘magic bullet’ and ease of accessibility (not prescribed by a physician).^18^ In addition, advertisements that highlight inflated claims, such as “fast-acting” or “natural” can affect purchasing behavior.^18^

Given the role that magazines play in providing health and nutrition information and the extent to which readers utilize magazines for this purpose, it is of interest to evaluate their content.

There is substantial evidence that print magazines have a negative influence on body image and disordered eating behavior particularly in adolescent girls who are generally more at risk for eating disorders and youth and adults who are already exhibiting disordered eating cognition and behaviors.^13,19-23^ This study’s aim was therefore to assess the prevalence and type of weight loss information found in articles and advertisements within mainstream women’s magazines that were specific to health and fitness. In addition, we sought to identify the prevalence of selected themes used within the magazines’ articles and advertisements when promoting weight reduction.

## Materials and Methods

This cross-sectional study’s sample consisted of 31 hard copy issues from 5 popular US women’s health and fitness-oriented magazines. The magazines utilized for data collection in this study were as follows: *Fitness, Health, Self, Shape*, and *Women’s Health* and were selected for their primary focus on women’s health and fitness as well as their wide reach with a combined readership of 36 300 000.^24^ The mean age of readers is 43 years old.^24^

Magazine issues used for analysis covered a span of seven months (March to September 2013). These months were selected to span seasons in addition to the summer months when we hypothesized weight reduction advertisements may be more common. Of note, *Fitness* magazine is released bi-monthly, therefore an issue for each month was not available. All magazine pages were reviewed except for the front cover as advertisements and articles do not appear there. A coding sheet was adapted based on a prior study focused on skin advertisements and editorials and was tailored to capture relevant content related to weight loss.^25^ Articles related to weight were identified in each magazine’s Table of Contents. Articles were determined to be related to weight if they included a theme (or themes) identified in Table 1.

**Table 1:** Weight reduction articles by topic in *Fitness, Health, Self, Shape, and Women’s Health*, March 2013 through September 2013

**Topics of weight reduction articles**	**n (%)**	**Range**
Avoid being flabby	8 (1.7)	0-4
"Baby" fat	8 (1.7)	0-3
Being satisfied with how one looks	10 (2.1)	0-2
Burning calories	41 (8.7)	0-5
Dieting/eating less/food choices	88 (18.6)	0-37
Eating healthy/nutritious food options/balanced meals	40 (8.5)	0-19
Exercising/exercise plans and workouts	151 (32.0)	0-19
Healthy ways to lose weight	23 (4.9)	0-2
Keeping weight off	14 (3.0)	0-2
Losing weight for health reasons	30 (2.1)	0-5
Losing weight to please oneself	14 (3.0)	0-2
Losing weight to please others	1 (0.2)	0-1
Quick weight loss	13 (2.8)	0-5
Other	31 (6.6)	0-8

Themes portrayed to the reader within each article were assessed and recorded as they related to weight reduction. Once the article was identified in the Table of Contents, the article was located in the magazine and its content was reviewed. The coding sheet was used to record weight loss theme(s) present in each article. The coding sheet allowed for enumeration and classification of weight loss product advertisements as well. Advertisements were included in the sample if they were a recognized paid source from a company. “Products of the month” based on editors’ selection were excluded. This decision was made in order to streamline coding and adhere to our objective of analyzing advertisements (versus endorsements).

Weight loss products identified in advertisements were categorized as fat blockers, fat burners, general weight loss pills, hunger reduction strategies, and “other” (cleanse/burn, combo pilling, programs, shakes/drinks). For each weight loss product advertisement coded, the following themes common to weight loss were identified: achievement, “before and after,” happiness, sex appeal, and “other.”

A single researcher coded the magazines (A.B.) by using the methodology explained above. A 20% sample of magazines issues was recoded by the same coder to determine intra-rater reliability which establishes the degree of agreement among repeated administrations of the coding instrument performed by a single coder. Cohen’s kappa, which takes into account the amount of agreement that could be expected to occur due to chance, was used to assess intra-rater reliability and found to be excellent (k = 0.90). Frequency distributions for (1) the number of articles devoted to weight reduction, (2) weight loss themes identified in these articles, (3) number of weight loss advertisements and products displayed, and (4) type of products and themes portrayed in the advertisements were calculated along with the range of items per magazine issue. Data were analyzed using IBM SPSS, version 22.

## Results

Among the 31 issues of the five women’s fitness magazines examined, a total of 819 articles and 2073 advertisements were reviewed. Of these, 39 articles (4.8%, 95% CI = 3.3% to 5.5%) were related to weight loss and covered 14 different topics related to weight reduction with 472 mentions of these topics in the articles (Table 1). The majority of topics in the articles addressed exercising, exercise plans and workouts (32%, 95% CI = 28.8% to 33.6%) followed by dieting/eating less/food choices (18.6%, 95% CI = 15.9% to 19.9%). Burning calories and eating nutritious foods/having balanced meals (8.7%, 95% CI = 6.7% to 9.6% and 8.5%, 95% CI = 6.6% to 9.5% respectively) accounted for a small fraction of the topics of the weight reduction articles. Other less commonly addressed topics included being satisfied with how one looks (2.1%, 95% CI = 1.1% to 2.6%) and how to avoid being flabby (1.7%, 95% CI = 0.8% to 2.1%). In addition, few of the topics addressed losing weight for health reasons (6%, 95% CI = 4.5% to 6.9%) or specifically emphasized healthy ways to lose weight (4.9%, 95% CI = 3.4% to 5.6%).

We identified 87 advertisements for weight loss products (4% of 2073 advertisements, 95% CI = 2.7% to 4.7%). Of those products advertised, general weight loss pills were found to be the most commonly promoted products (46%, 95% CI = 42.6% to 47.7%) (Table 2). Fat burners were also frequently advertised (14.9%, 95% CI = 12.5% to 16.1%) followed by hunger reduction strategies (10.3%, 95% CI = 8.2% to 11.3%) and fat blockers (6.9%%, 95% CI = 5.2% to 7.8%). Obtaining a sense of achievement (25.2%, 95% CI = 22.2% to 26.6%) was the most commonly identified theme among the weight loss product advertisements. An emphasis on “before and after” weight loss (20.7%, 95% CI = 18.0% to 22.1%) was second most common. Fewer advertisements emphasized happiness (9.0%, 95% CI = 7.0% to 9.9%), natural weight loss (9.0%, 95% CI = 7.0% to 9.9%), and sex appeal (7.2%, 95% CI = 5.4% to 8.1%).

**Table 2:** Weight loss advertisements by product type and advertisement theme in *Fitness,* Health, Self, Shape, and Women’s Health, March 2013 through September 2013

	**n (%)**	**Range**
Products	87	0-7
Fat blocker	6 (6.9)	0-2
Fat burners	13 (14.9)	0-3
General weight loss pills	40 (46.0)	0-7
Hunger reduction strategies	9 (10.3)	0-2
Weight loss programs	8 (9.2)	0-1
Other	11 (12.6))	0-2
Cleanse/cleanse and burn	3 (27.3)	0-1
Combo pilling	5 (45.5)	0-1
Shake/drink	3 (27.3)	0-1
Themes	111	0-7
Achievement	28 (25.2)	0-5
"Before and after"	23 (20.7)	0-5
Happiness	10 (9.0)	0-2
Sex appeal	8 (7.2)	0-2
Natural weight loss	10 (9.0)	0-1
Other	32 (28.8)	0-7
Delicious tasting	3 (9.4)	0-1
Easy and simple plan/product	7 (21.9)	0-1
Fast acting	3 (9.4)	0-1
For women specifically	4 (12.5)	0-1
Powerful	5 (15.6)	0-1
Miscellaneous other	10 (31.3)	0-1

## Discussion

Approximately 5% of the articles in our sample from the five leading women’s mainstream health and fitness magazines in the United States were devoted to weight reduction. There is an opportunity for this genre to communicate frequent, accurate messages about healthy weight loss. In our sample, the majority of the weight loss-related editorial content emphasized exercise and workouts. While themes such as healthful approaches to eating and losing weight for health reasons were also noted, they appeared less frequently. These findings are consistent with findings from Willis and Knobloch-Westerwick’s recent study which analyzed roughly 5000 pages of editorial-related content in health and fitness magazines.^15^

Because the pervasive nature of advertising and marketing has been well established, our findings related to advertising are of particular concern. With 4% of advertisements aimed at weight reduction, the most common category of advertisements we identified was for weight loss pills (46%). As such, these advertisements may emphasize that weight loss can happen quickly. The Centers for Disease Control and Prevention (CDC) cautions that, in order to achieve related health benefits, those seeking to lose weight should not do so rapidly.^26^ The CDC also advises that those who lose weight gradually are more likely to maintain their weight loss. Modest weight loss is recommended to decrease risk of developing obesity-related chronic diseases.^26^ Themes commonly noted in the advertisements for weight loss products appealed to appearance-based motivations that may have deleterious effects on women’s weight loss perceptions and behaviors. These themes included feeling a sense of achievement and “before and after” photographs.^17^ While these messages may appeal to a reader concerned about losing weight, advertisements of this nature can often be misleading and promote unrealistic results.^5^ They may also perpetuate readers’ negative self-concept when comparing perceived personal weight loss “failures” with advertised themes of success and achievement.

The results of this study are of particular concern considering that weight loss products account for the highest proportion of fraud claims to the FTC.^27^ According to Consumer Reports, “Since 2010, the FTC has collected nearly $107 million in consumer restitution for deceptive weight-loss claims.”^3^ In light of these recent settlements, the FTC has updated media standards with regard to the advertisement of weight loss products. These guidelines highlight selected wording that should help consumers and media outlets better identify fraudulent weight loss products.^28^ Further research is needed to determine whether these standards have influenced consumers’ perceptions and purchasing behaviors related to these products.

This study is limited by its cross-sectional design and sample size. Naturally, lengthening the period of assessment would provide a more comprehensive overview regarding trends in content and products related to weight loss. However, we purposefully selected a sample of health and fitness-focused magazines with a large, combined readership. This is the first study we are aware of that explores both the prevalence of weight loss-related articles and advertisements and assesses themes in both within this particular magazine genre. These magazines are likely purchased with the intent of gathering health information and strategies related to weight loss and body shaping. Our findings suggest that these magazines can be more proactive in promoting healthy weight loss themes presented in their articles’ content and by their advertisers. They can, for instance, be more selective about advertising products and strategies that focus on healthful eating and exercise behaviors that are supported by the scientific literature. In addition, advertised products and article themes should align with and promote weight loss goals that are obtainable and sustainable. Given their extensive reach, American health and fitness-focused magazines are in a position to (1) limit advertisements that may include misleading claims and (2) communicate accurate messaging about healthy, achievable, and sustainable weight loss.

## Ethical approval

The Institutional Review Boards at William Paterson University, Lehman College, and Columbia University deemed this study not human subjects research.

## Competing interests

The authors declare that they have no conflict of interest.

## Authors’ contributions

DE conceptualized the study, designed data collection methodology, and led manuscript development. CB assisted in conceptualizing the study, designing data collection methodology, and manuscript development. GH conducted data analysis and assisted with manuscript development. AB was responsible for data collection and assisted with manuscript development. MH assisted with interpreting the study’s findings and manuscript development.

## References

[1] National Institute of Diabetes and Digestive and Kidney Diseases. Overweight and obesity statistics. http://www.niddk.nih.gov/health-information/health-statistics/Pages/overweight-obesity-statistics.aspx. Accessed 3 March 2016.

[2] Nutrition & weight management. Boston Medical Center website; 2014. http://www.bmc.org/nutritionweight/services/weightmanagement.htm. Accessed 29 June 2014.

[3] Don’t risk your money and health on bogus weight-loss products. Consumer Reports website; 2014. http://www.consumerreports.org/cro/news/2014/06/don-t-risk-your-money-and-health-on-bogus-weight-loss-products/index.htm. Accessed 1 July 2014.

[4] Volpe SL (2006). Popular weight reduction diets. J Cardiovasc Nurs.

[5] Deception in weight-loss advertising workshop: seizing opportunities and building partnerships to stop weight-loss fraud. Federal Trade Commission website; 2003. http://www.ftc.gov/sites/default/files/documents/reports/deception-weight-loss-advertising-workshop-seizing-opportunities-and-building-partnerships-stop/031209weightlossrpt.pdf. Accessed 28 June 2014.

[6] Cutilli CC (2010). Seeking health information: what sources do your patients use?. Orthop Nurs.

[7] Maibach EW, Weber D, Massett H, Hancock GR, Price S (2006). Understanding consumers’ health information preferences: Development and validation of a brief screening instrument. J Health Commun.

[8] Dutta-Bergman MJ (2004). Primary sources of health information: comparisons in the domain of health attitudes, health cognitions and health behaviors. Health Commun.

[9] Botta RA (2003). For your health? the relationship between magazine reading and adolescents’ body image and eating disturbances. Sex Roles.

[10] Harper B, Tiggemann M (2008). The effect of thin ideal media images on women’s self-objectification, mood, and body image. Sex Roles.

[11] Tiggemann M (2003). Media exposure, body dissatisfaction and disordered eating: television and magazines are not the same!. European Eat Disorders Rev.

[12] Turner SL, Hamilton H, Jacobs M, Angood LM, Dwyer D (1997). The influence of fashion magazines on the body image satisfaction of college women: an exploratory analysis. Adolescence.

[13] Thomsen SR, Weber MM, Brown LB (2001). The relationship between health and fitness magazine reading and eating-disordered weight-loss methods among high school girls. Am J Health Educ.

[14] Thomsen SR, Bower DW, Barnes MD (2004). Photographic images in women’s health, fitness, and sports magazines and the physical self-concept of a group of adolescent female volleyball players. J Sport Soc Issues.

[15] Willis LE, Knobloch-Westerwick S (2014). Weighing women down: messages on weight loss and body shaping in editorial content in popular women’s health and fitness magazines. Health Commun.

[16] Putterman E, Linden W (2004). Appearance versus health: Does the reason for dieting affect dieting behavior?. J Behav Med.

[17] Geier AB, Schwartz MB, Brownell KD (2003). “Before and after” diet advertisements escalate weight stigma. Eat Weight Disord.

[18] Saper RB, Eisenberg DM, Phillips RS (2004). Common dietary supplements for weight loss. Am Fam Physician.

[19] Utter J, Neumark-Sztainer D, Wall M, Story M (2003). Reading magazine articles about dieting and associated weight control behaviors among adolescents. J Adolescent Health.

[20] van den Berg P, Neumark-Sztainer D, Hannan PJ, Haines J (2007). Is dieting advice from magazines helpful or harmful? Five-year associations with weight-control behaviors and psychological outcomes in adolescents. Pediatrics.

[21] Vaughan KK, Fouts GT (2003). Changes in television and magazine exposure and eating disorder symptomatology. Sex Roles.

[22] Hamilton K, Waller G (1993). Media influences on body size estimation in anorexia and bulimia. An experimental study. Br J Psychiatry.

[23] Shaw J (1995). Effects of fashion magazines on body dissatisfaction and eating psychopathology in adolescent and adult females. Eur Eat Disord Rev.

[24] MRI+ website. http://www.mriplus.com/publications/index.aspx. Accessed 25 April 2014. Published 2003.

[25] Basch CH, Hillyer GC, Basch CE (2013). Descriptive analysis of articles and advertisements pertaining to skin care and skin cancer prevention in two popular parenting magazines, 2000-2010. Prev Chronic Dis.

[26] Healthy Weight - it’s not a diet, it’s a lifestyle! Centers for Disease Control (CDC) website. http://www.cdc.gov/HEALTHYWEIGHT/LOSING_WEIGHT/INDEX.HTML.Accessed 26 June 2014.

[27] Consumer fraud in the United States, 2011: staff report of the bureau of economics federal trade commission. Federal Trade Commission website; 2013. http://www.ftc.gov/sites/default/files/documents/reports/consumer-fraud-united-states-2011-third-ftc-survey/130419fraudsurvey_0.pdf. Accessed 26 June 2014.

[28] Gut check: a reference guide for media on spotting false weight loss claims. Federal Trade Commission website; 2014. http://www.business.ftc.gov/documents/0492-gut-check-reference-guide-media-spotting-false-weight-loss-claims. Accessed 26 June 2014.

